# Sensitivity of Ultrasonic Coda Wave Interferometry to Material Damage—Observations from a Virtual Concrete Lab

**DOI:** 10.3390/ma14144033

**Published:** 2021-07-19

**Authors:** Claudia Finger, Leslie Saydak, Giao Vu, Jithender J. Timothy, Günther Meschke, Erik H. Saenger

**Affiliations:** 1 Fraunhofer IEG, Fraunhofer Research Institution for Energy Infrastructures and Geothermal Systems, Am Hochschulcampus 1, 44801 Bochum, Germany; erik.saenger@hs-bochum.de; 2 Institute for Geology, Mineralogy and Geophysics, Ruhr-University Bochum, Universitätsstrasse 150, 44801 Bochum, Germany; leslie.saydak@rub.de; 3 Reservoir Engineering and Rock Physics, Bochum University of Applied Sciences, Am Hochschulcampus 1, 44801 Bochum, Germany; 4 Institute for Structural Mechanics, Ruhr-University Bochum, Universitätsstrasse 150, 44801 Bochum, Germany; thi.vu-h6d@ruhr-uni-bochum.de (G.V.); timothy.jithenderjaswant@rub.de (J.J.T.); guenther.meschke@rub.de (G.M.)

**Keywords:** damage detection, concrete-like structures, coda waves, ultrasound, wave propagation, discrete element modeling, sensitivity study

## Abstract

Ultrasonic measurements are used in civil engineering for structural health monitoring of concrete infrastructures. The late portion of the ultrasonic wavefield, the coda, is sensitive to small changes in the elastic moduli of the material. Coda Wave Interferometry (CWI) correlates these small changes in the coda with the wavefield recorded in intact, or unperturbed, concrete specimen to reveal the amount of velocity change that occurred. CWI has the potential to detect localized damages and global velocity reductions alike. In this study, the sensitivity of CWI to different types of concrete mesostructures and their damage levels is investigated numerically. Realistic numerical concrete models of concrete specimen are generated, and damage evolution is simulated using the discrete element method. In the virtual concrete lab, the simulated ultrasonic wavefield is propagated from one transducer using a realistic source signal and recorded at a second transducer. Different damage scenarios reveal a different slope in the decorrelation of waveforms with the observed reduction in velocities in the material. Finally, the impact and possible generalizations of the findings are discussed, and recommendations are given for a potential application of CWI in concrete at structural scale.

## 1. Introduction

The assessment of the residual safety and durability of reinforced concrete structures (bridges, buildings, etc.) is vital for the efficient planning of retrofitting strategies for modern-day infrastructure networks. Ultrasonic measurements are frequently used to monitor the structural health of such concrete structures with the objective of detecting changes as early as possible before devastating damages occur.

Due to multiple scattering effects in heterogeneous media, the coda wave, i.e., the trailing diffuse part of wave signals, is especially sensitive to small changes in the apparent seismic velocity [1,2]. In addition to structural damage caused, i.e., by cracking, changes of the stress state [3], chemical alterations [4], and temperature and moisture changes [5] were identified as observable causes for these relative velocity changes dv/v. Recently, Coda Wave Interferometry (CWI) [2,6] has been identified as superior for estimating dv/v in reservoir rocks [6,7], in concrete sample specimens [2] and in larger concrete structures [8,9]. Furthermore, the possibility to monitor localized velocity changes and to generate tomographic images of load-induced damages has been demonstrated [10].

In addition to ultrasonic laboratory experiments on different-sized concrete specimen, numerical simulations have been used to explain and understand the coda signal and its sensitivity to various damage geometries [1,11]. In this study, realistic numerical three-dimensional concrete specimens are created using a concrete mesostructure generator [12] to account for realistic volume fractions of aggregates of different sizes embedded into a mortar matrix. Three-dimensional computational concrete models using the Discrete Element Method [13] are subjected to tensile loading to produce a series of structural models with evolving cracks. An ultrasonic wavefield is simulated in these models using a finite-difference simulator [14]. The source signal emitted by a commonly used ultrasonic transducer is simulated to imitate classical ultrasonic laboratory experiments as close as possible. The sensitivity of Coda Wave Interferometry (CWI) to different damage scenarios is investigated systematically by changing the input models. In particular, models characterized by diffuse changes of the microstructural damage conditions, such as induced by temperature or moisture variations, and localized structural damage, are compared. CWI is performed using the stretching method [2,6] to estimate the apparent relative velocity change dv/v. Comparing dv/v to the (de-) correlation of the waveforms reveals that localized damages and global velocity changes can be discriminated using CWI. Finally, the ability to upscale the results to large concrete structures is discussed. This study presents a realistic numerical analysis of CWI. As coda signals are highly sensitive to the environmental effects (e.g., temperature, and moisture), it is still a significant challenge to reliably identify and classify damage in concrete [15]. In this regard, numerical simulations can be employed in addition with ultrasonic laboratory experiments to explain and understand the coda signal and its sensitivity to various damage types. The ability of numerical simulations to tune the parameters and damage scenarios, as well as to exclude environmental conditions offers an effective tool to analyze and interpret the change of CWI features with respect to the damage evolution.

## 2. Materials and Methods

To study the effect of different damage scenarios on ultrasonic wave propagation and the analysis with CWI, simulations of the full elastic seismic wavefield were performed using a rotated staggered-grid finite-difference scheme [14]. All simulations were performed in a three-dimensional model space with realistic structural models obtained from discrete element modeling (DEM) [13]. Synthetic sources were calibrated to closely resemble the source signal emitted by common ultrasonic transducers. The following sections outline the method used to build a numerical model, simulate the ultrasonic wave propagation and analyze the results using coda wave interferometry.

### 2.1. Generation of Realistic Numerical Concrete Models

At the mesoscale, the internal material structure of concrete is characterized by a large volume fraction of aggregates (up to 60–70%) of different sizes, embedded into a mortar matrix. Subjected to an external load, the mesoscopic heterogeneity in concrete contributes to highly complicated stress- and ultrasonic wave fields. Especially when interacting with the initial defects at finer scales, it results in a complex damage process ranging from diffuse microcracking to localized fracture [16]. Thus, to realistically simulate the fracture process in concrete, it is of importance to realistically take into account the spatial distribution of aggregates. To this end, a concrete mesostructure generator (CMG) was developed [12] that allows an efficient computation of voxel-based synthetic concrete numerical samples. The algorithm can generate realistic concrete mesostructures given the aggregate size distribution. In what follows, a summary of the concrete mesostructure generator is provided, for details we refer to [17]. A Python implementation is available at https://pycmg.readthedocs.io/en/latest/ accessed on 15 July 2021.

#### 2.1.1. Generation of Synthetic Aggregates

In general, a concrete aggregate can be characterized by its multiple faces, sharp corners/edges, and multiple non-convex surfaces. In our work, aggregates are modeled by means of irregular polyhedrons with concave depressions. The generation of a virtual aggregate involves the reduction of an initial cuboid to the polyhedron by applying multiple random cuts tangential to an imaginary inscribed ellipsoid. The ellipsoid geometry is a function of the maximum size and the elongation ratio (ratio of the longest dimension to the shortest dimension). The random orientation of an aggregate is realized by applying rotational transformation with respect to the global axes and the concave depressions are introduced in terms of a 2D Gaussian surface at random locations lying on the surface of the inscribed ellipsoid.

#### 2.1.2. Assembly Algorithm

After each aggregate or grain is generated, the next step is to embed it into a prescribed hosting mortar matrix (note that in the current paper, the terms aggregate and grain are used interchangeably). The main objective of the assembly step is to randomly pack aggregates that follow the statistical particle size distribution (PSD) of concrete, while fulfilling the non-overlapping condition between aggregates. The packing strategy begins with the pre-processing of input packing parameters such as the grading curve of concrete, and the total volume fraction. Each particle family is sorted according to their size and the largest particles are placed first, followed by smaller particles until the target volume fraction of each size is reached. The current aggregate is successfully embedded into the mesostructure only if it does not intersect with other aggregates as well as the number of attempts does not exceed an a priori defined value. The mesostructure is periodic. This is achieved by assembling the part of the aggregate lying outside the surface of the mesostructure domain to the opposite surface. This feature allows the generation of large-sized concrete models, for instance, at the structural scale.

#### 2.1.3. Variation of Models

**Figure 1:**
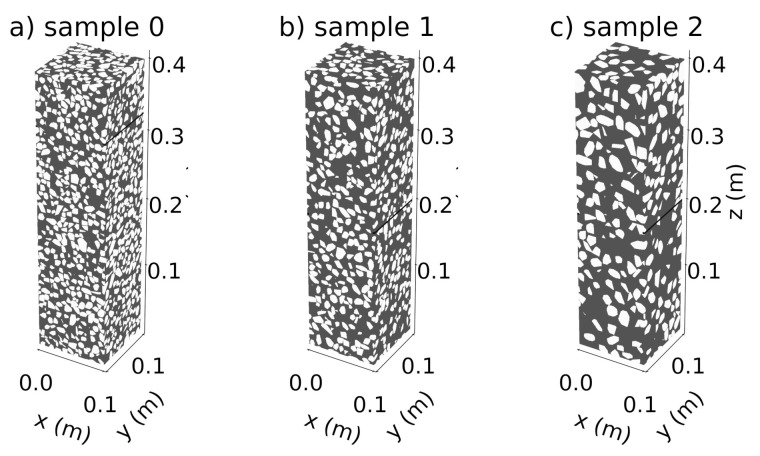
Three-dimensional models of concrete specimens with different grain sizes used in this study. Dark Grey: mortar matrix and white: aggregates.

**Figure 2:**
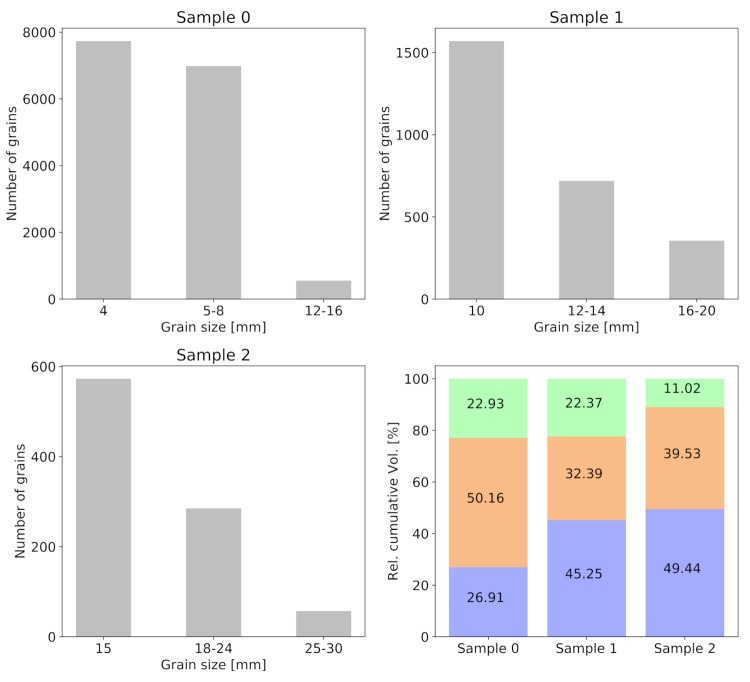
Grain count of samples 0, 1, and 2 with respect to the grain size groups. Bottom-right: Relative volume fraction of each aggregate group in samples 0, 1, 2. For instance, sample 0 is made up of 26.91% grain size 4 mm (group 1), 50.16% of grain size of 5–8 mm (group 2), and 22.93% of grain size 12–16 mm (group 3).

In the current study, we considered three concrete mesostructures with three different grain size ranges. The goal is to take into account the influence of grain size on the simulated Coda signals with increasing damage level. The three samples are denoted as sample 0, sample 1, sample 2 (Figure 1) generated according to the aforementioned procedure. The grain size distribution for the three samples is listed in Table 1, with sample 0 (2) having the highest (smallest) packing density. For a given concrete sample, aggregates are grouped into three grain families and particle count per grain family were recorded and presented in Figure 2. Additionally, Figure 2 bottom-right plots the relative grain volume fractions of the 3 samples.

### 2.2. Numerical Modeling of Damage in Concrete Subjected to Uniaxial Tension

With the concrete mesostructures at hand, we proceed to model damage of concrete using the Discrete Element method (DEM). In DEM, a granular material (e.g., concrete, rock) is described as a packing of DEM-particles that are linked together by cohesive frictional forces. Within the material, the induced forces are transmitted via a contact network between particles and the dynamics of these particles is governed by Newton’s second law [13]. The contact network is first established and updated by identifying the DEM-particles and nearest neighbor interactions. The interaction forces are evaluated based on the relative displacements in the current DEM-particle configuration. Next, the resultant interaction forces are used in conjunction with the applied external forces as input for the equations of motion in the time integration step to solve for the new position of all DEM-particles.

The inter-particle modeling in the DEM approach based on [18] is briefly summarized. Concisely, the interaction of the DEM-particles under tension in normal direction is governed by a damage softening law and the tangential behavior of contact between the DEM-particles follows the modified Mohr–Coulomb frictional law. Details of contact description can be found in [19]. The calibrated parameters of mortar and aggregate materials are listed in Table 2. The parameter $\frac{\varepsilon_{f}}{\varepsilon_{0}}$ controls the ductility of materials in the post-peak regime. To study the role of microcracking on the simulated Coda signals, the ductility parameter of mortar and aggregates is set to 5 in DEM sample 0 to simulate brittle damage with minimal evolution of microcracks before localized tensile failure of the specimen occurs. In contrast, the ratio $\frac{\varepsilon_{f}}{\varepsilon_{0}}$ is set to 50 for samples 1 and 2 to provoke a more ductile characteristic and an extended microcracking process in the prepeak regime.

**Figure 3:**
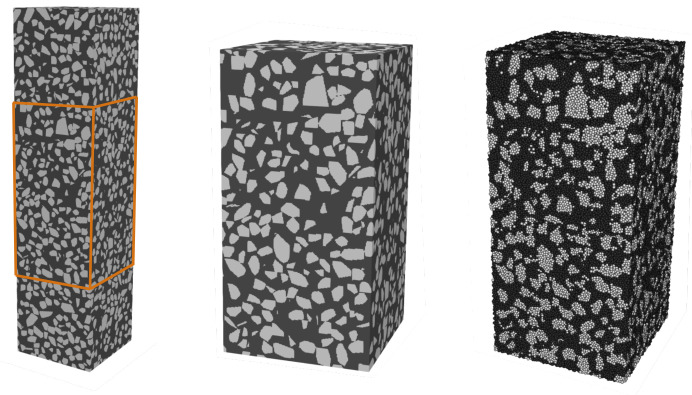
Model generation for concrete sample 0. **Left**: Voxelised sample including the region, where damage is expected (orange box) (**left**), **center**: enlargement of the potential damage region **right**: DEM discretization of the potential damage zone ($a_{min}$ = 4mm, $a_{max}$ = 16mm) considered in the uniaxial tension simulation (black: mortar phase, grey: aggregate phase).

The modeling of damage in concrete begins with the generation of a random densely packed ensemble of 661,830 DEM-particles with radius of 0.8mm (Figure 3 Left). Next, the voxelised mesostructure generated in the previous section are used to assign material properties of DEM-particles. In all DEM simulations, only the center region of the specimen between Z=0.1andZ=0.3m (in Figure 1) of the voxelised mesostructure is considered and denoted as “potential damage zone”. Notches of 3mm depth were introduced as artificial imperfections on two faces at mid-height of the numerical samples to prevent multiple cracks and to enforce the primary crack location, as suggested in [20]. Following the approach outlined in [21], DEM uniaxial tension test is simulated as follows: The displacement control uniaxial tension test is simulated by prescribing a constant velocity of 1mm/s on three layers of particles at the top surface, while the position of particles at the bottom face is fixed. To ensure numerical stability and a fast convergence of the dynamics DEM model to a static response, a numerical damping is set to 0.2 and an average time increment is specified at $2.44\times 10^{-7}\;s$. In this work, DEM simulations were performed using an open-source DEM software ‘WooDEM’ [22]. The software is written in C++11 and supports OpenMP parallelization. The computational time required for the simulation of the confined compression test on these above DEM sample is approximately 15 h. Each simulation is performed on 20 IntelR^©^XeonR^©^Gold 6148 CPUs running in parallel at 2.40GHz.

**Figure 4:**
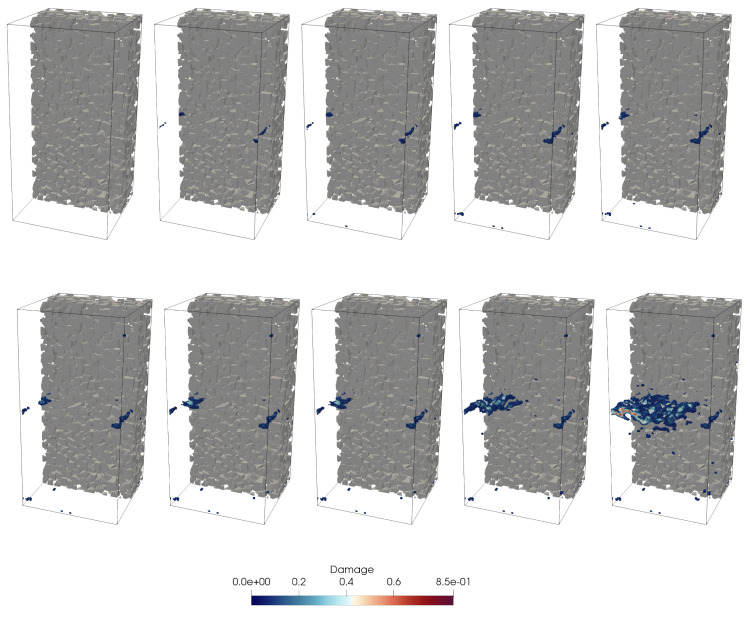
Computed damage evolution in concrete sample 0 subjected to uniaxial tension. For the visualization of the crack topology, only damaged mortar with d > 7% is shown. Solid voxels in grey color represent densely packed aggregates.

At each time increment, the damage parameter *d* of particle *i* is evaluated by averaging the damage variable over all cohesive contacts associated with that particle. Particles/voxels with parameter of value 0 are in undamaged stage, while d=1 indicates a fully damaged stage. The damage field serves as input to set the material parameters required in the successive wave propagation simulations. In particular, the local effective (degraded) stiffness is computed by scaling the initial stiffness by the damage factor (1−d). Figure 4 shows a realistic crack pattern obtained from the DEM simulation of sample 0. The models and crack distribution are similar to those obtained by imaging concrete specimens in laboratory experiments [23].

For each numerical sample, ten mesostructure snapshots at different damage levels are extracted for the wave propagation analysis. Figure 4 illustrates the evolution of cracks in sample 0. A summary of tensile behavior of three DEM concrete samples can be found in Table 3.

### 2.3. Preparation of Three-Dimensional Mesostructure Models for Ultrasonic Wave Propagation Simulations

To simulate the ultrasonic wave field, the mesostructural models for concrete specimen described in the previous section are assigned appropriate parameters for P- and S-wave velocity and density. These parameters for the initially undamaged state are listed in Table 4. For reference, also the elastic bulk moduli and shear moduli are provided. Standard elastic parameters of cement are assigned to the mortar matrix [24] to simulate a homogeneous cementitious matrix and elastic parameter of basalt are assigned to the grains.

The virtually generated mesostructure models (for three different aggregate compositions denoted by sample 0, 1, and 2) are subjected to tensile loading. Each of the samples 0, 1 and 2, subjected to uniaxial tension was analyzed by the DEM up to failure, covering the complete damage regime, from the intact state, to an initial diffuse growth of microcracks up to complete failure of the sample. For the wave propagation analysis described in the next subsection, local elastic velocities are assigned at the corresponding locations according to the local damage level *d*. This first set of models is denoted as “macro+microcrack” for the remainder of this study.

A second set of models has been generated for samples 1 and 2 in which, for the wave propagation simulations, diffuse damage, i.e., damage levels corresponding to a lower-level load (d<0.25) are suppressed, and therefore only more significant damage (“macrocracks”) is considered for the wave propagation analysis. The purpose of this second set is to evaluate the sensitivity of CWI regarding the ability to detect microcracking of concrete in earlier loading stages as compared to macrocracks. This set of models is denoted “macrocrack”.

A third set of models has been generated, in which only the mortar matrix, without basalt grains, is simulated. This model is assigned elastic parameters according to the DEM models for sample 0 and serves to investigate the influence of the presence of grains in the model. This set of models is denoted as “homogeneous”.

A final set of models has been generated with the initially intact mesostructures for sample 0, sample 1 and sample 2. Instead of using the DEM simulated models, the P- and S-wave velocities have been manually decreased stepwise in the complete model. This set of models simulates environmental effects acting on the complete concrete specimen. The velocity reduction has been done separately for the matrix phase (“diffuse damage (matrix)”) and the grain phase (“diffuse damage (grains)”).

In the following sections, the models described above are denoted as their aggregate compositions (“sample 0”, “sample 1”, “sample 2”, “homogeneous”) plus their history of damage (“micro+macrocrack”, “macrocrack”, “diffuse damage”).

### 2.4. Simulation of Ultrasonic Waves in a Three-Dimensional Elastic Medium

Ultrasonic wave field simulations were performed with a model size of 0.4m×0.1m×0.1m (Figure 1). The concrete models are surrounded on each side with a layer of vacuum that is two grid points thick. In this layer, the velocities are set to zero while the density is set to a small but non-zero value to take into account that the concrete specimen are surrounded by air.

In this study, a rotated staggered-grid finite-difference scheme [14] is used to propagate the ultrasonic waves through the realistic numerical concrete models. The rotated staggered-grid has the distinct advantage of distributing the modeling parameters in an elementary cell in such a way that components of the same physical property are located at one single position. Therefore, no averaging of the elastic moduli is needed making it especially well-suited for modeling the wave propagation in viscoelastic or highly heterogeneous media [26]. In this study, finite-difference operators with a length of two are used in time and in space.

The spacing between adjacent points of the evenly spaced finite-difference grid is $0.5\times 10^{-3}\;m$. With this grid spacing and the chosen velocities, frequencies of up to approximately 450kHz can be simulated with minimized numerical dispersion. The time increment was set to $5\times 10^{-8}\;s$ to ensure a stable simulation. The total simulation length is 80,000 timesteps=4ms and each simulation takes about 6h on three computing nodes.

To simulate the typical decline in amplitudes with time observed in the coda wave of ultrasonic measurements, a homogeneous intrinsic attenuation has been applied. At the central frequency of the source signal, the attenuation is $Q_{p}\approx 360$.

**Figure 5:**
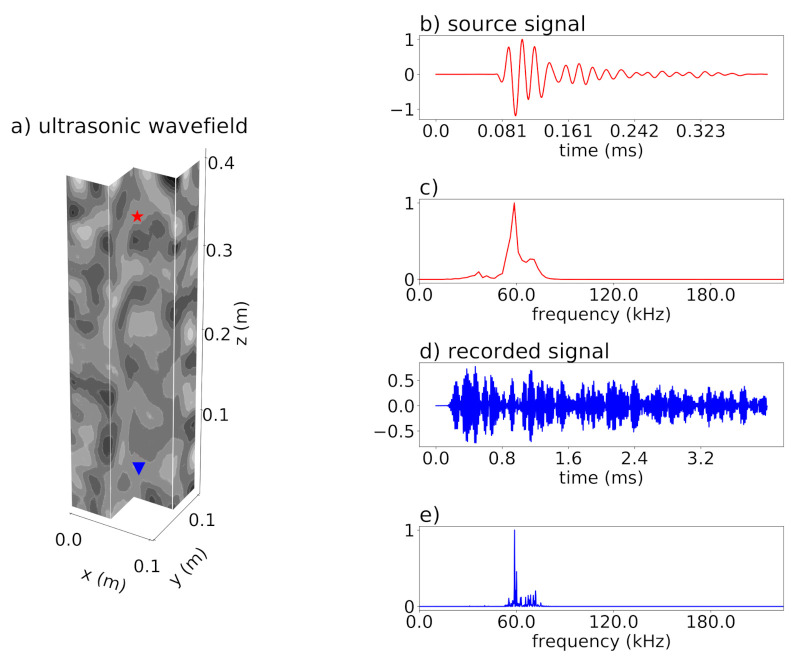
(**a**) Three-dimensional ultrasonic wave field after 0.5ms simulation time (sample 0) with source (red star) and receiver (blue triangle) positions. Z component of displacement is shown in grayscale from largest positive value (black) to largest negative value (white). (**b**) Displacement in z-direction of the used realistic source signal. (**c**) Frequency content of the source signal. (**d**) Mean of the xx- and zz-stress components recorded at the receiver position. (**e**) Frequency content of the mean stress.

For all simulations, a single source and a single receiver are used. Source and receiver are located at the center of the x-y-slice and separated in z-direction by 0.3m (Figure 5a). The source is implemented as a six-component moment tensor source with only the xx- and zz-component being non-zero. This resembles the radiation characteristic of common ultrasonic transducers [27]. Furthermore, the source signal itself is calibrated using laboratory measurements of transducer signals (Figure 5b,c). The central frequency of the source signal is fc=60kHz. The grains in the three models are therefore smaller than half the dominant wavelength (Table 5).

For the analyses in this study, the xx- and zz-component of the stress recorded at the receiver position are averaged (Figure 5d,e) and used as the input for coda wave interferometry. This produces a trace that closely resembles the recordings of a direction-dependent transducer. For comparable acoustic emission traces, please refer to e.g., [24]. For most of the study, the complete recorded trace is used, including the first arrival.

Since transducers have a discrete physical size, preliminary tests have been performed with implementing multiple sources in a localized area. The results did not show a significant difference as compared to the implementation of a single point source. Therefore, sources are implemented as point sources in this study.

### 2.5. Coda Wave Interferometry (CWI)

Following the approach outlined in [2], the stretching method is used to compute the relative apparent velocity change dv/v of the medium after it was perturbed. The stretching method is based on the principle that a change in the medium’s elastic properties results in a linear phase shift between the wave fields $u_{u}$ and $u_{p}$ traveling through an unperturbed and perturbed medium, respectively [2]:

(1) $$u_{p}\left(t^{\prime }\right)=u_{u}\left(t^{\prime }\cdot \left(1+\Delta \right)\right),$$

with $t^{\prime }$ being the time of the recorded waveforms and Δ the dilation rate. By finding the Δ that results in the best fit between $u_{u}$ and $u_{p}$, the relative velocity change dv/v can be found. The best fit is determined by computing the cross-correlation coefficients CC between the unperturbed wave field (reference signal) $u_{u}$ and the perturbed wave field $u_{p}$ [2]:

(2) $$CC\left(t,\Delta \right)=\frac{\int_{t-T}^{t+T}u_{u}\left(t^{\prime }\right)u_{p}\left(t^{\prime }+\Delta \right)\;dt^{\prime }}{\sqrt{\int_{t-T}^{t+T}u_{u}^{2}\left(t^{\prime }\right)\;dt^{\prime }\int_{t-T}^{t+T}u_{p}^{2}\left(t^{\prime }\right)\;dt^{\prime }}},$$

with *t* representing the discrete simulation steps performed using the input models from each of the sets described in the previous section, *T* is the total recording time per simulation step and $t^{\prime }$ is the length of the recordings for the current simulation step. Each of the sets of mesostructural models described in the previous subsection is investigated separately to estimate the sensitivity of CWI to the different damage scenarios. The Δ where CC(t,Δ) is maximized ($CC_{max}\left(t\right)$) represents the apparent velocity change dv/v(t) for the analyzed simulation step. Multiple simulation steps with increased damage levels or decreased global velocities are analyzed for their apparent dv/v. To directly compare different damage scenarios, the Decorrelation Coefficient $DC=1-CC_{max}$ is used.

## 3. Results

Two main damage types are compared in this study: (1) Diffuse damage implemented by reducing velocities in the complete sample and (2) localized crack opening during increasing tensile loading computed by the DEM model. In the following, results from these scenarios are investigated separately first and then compared.

### 3.1. Diffuse Damage—Global Velocity Reductions

To simulate velocity changes corresponding to diffuse damage, i.e., induced by temperature or moisture variations, the P-wave and S-wave velocities have been reduced stepwise and the CWI workflow has been applied to every step. As described above, two scenarios are investigated in what follows: Reduction of velocities only in the matrix and reduction of velocities only in the grains.

**Figure 6:**
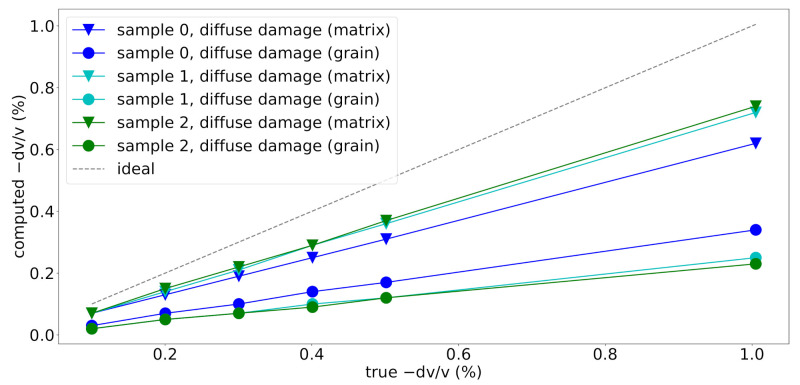
Observed velocity change against true velocity change (by manually reducing velocities in subsequent simulations) for each simulation step. Results are shown for samples 0, 1 and 2 for simulating diffuse damage in the mortar matrix and grain velocities.

The velocities were manually reduced in six discrete steps. A reduction in the velocities of the matrix phase results in observed velocity changes dv/v slightly lower than the true velocity changes (Figure 6). This discrepancy is because the velocities of the grains did not change. On the other hand, a reduction of the velocities in the grains, which occupy a smaller percentage of the volume of the model, results in underestimated velocity changes dv/v as well. The difference between the velocity change found for a reduction in the matrix and the grains reflects the total volume fractions occupied by grains in the three samples. For all cases, the linear trend introduced by a linear reduction in velocities can be captured very well.

A significant portion of the grains in sample 0 are smaller than 10% of the dominant wavelength used here (Table 5 and Figure 2) which has been reported as the theoretical resolution limit of CWI in optimal scenarios [28]. In this series of simulations no influence of the grain size on the CWI sensitivity has been observed.

### 3.2. Localized Damage—Uniaxial Tension Experiments

The numerical models of the concrete samples have been provided from the DEM simulations at different levels of uniaxial tensile loading and used for wave propagation simulations. The different loading levels correspond to different levels of damage and different locally reduced wave velocities of the mortar matrix.

**Figure 7:**
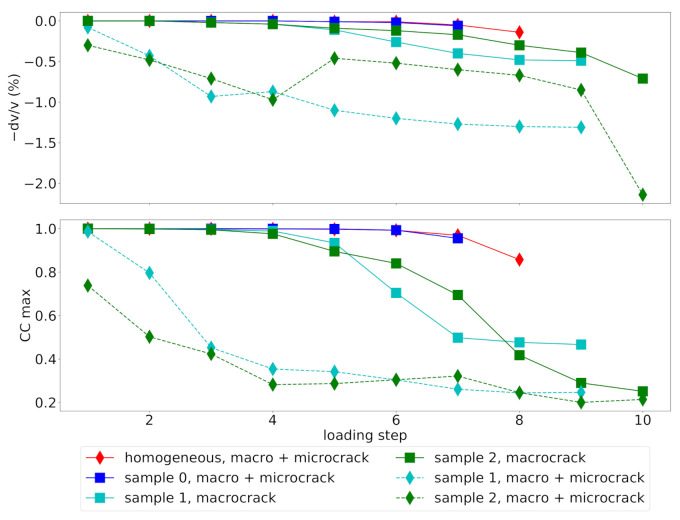
Evolution of apparent velocity change dv/v (**top**) and maximum cross-correlation coefficient $CC_{max}$ (**bottom**) with increasing loading steps obtained for the specimen models for mortar (“homogeneous”) and concrete samples 0,1 and 2 for the complete damage level range (“macro + microcrack”) and for artificially suppressed small damage levels, i.e., suppressed microcracks (“macrocrack”).

Comparing the observed velocity changes dv/v as a function of the loading step *t* (Figure 7) for different model sets illustrates the sensitivity of CWI to different damage scenarios. In addition to the three virtual concrete samples (0, 1, 2), also a mortar sample (denoted as “homogeneous”) has been investigated during all loading stages. As described in Section 2.3, for samples 1 and 2, two additional damage scenarios are investigated with suppressing small damage levels (d<0.25) and thus suppressing diffuse microcracking in early loading steps.

The initially homogeneous mortar specimen shows very brittle behavior and, consequently, almost no diffuse microcracking prior to a splitting crack leading to failure of the specimen. Hence, up to failure, only minimal velocity changes are recorded (red line in Figure 7). The damage evolution in sample 0 is also relatively brittle, with only little microcracks before failure, which is reflected by only a small velocity change throughout the loading. Since both sets use the same damage evolution modeled with DEM, the small difference is only due to the absence of the grains in the homogeneous model.

Samples 1 and 2 are more ductile and experience diffuse microcracking already in early loading stages. This is reflected by a considerable decay of $CC_{max}$ and dv/v already starting almost at the beginning of loading. However, when microcracking is suppressed by setting damage levels d<0.25 to the intact elastic parameters (solid lines in Figure 7, denoted “macrocrack”), the trend of dv/v and $CC_{max}$ is closer to the behavior of sample 0. This demonstrates that CWI can detect diffuse microcracks, which are reducing velocities in a larger volume of the model.

### 3.3. Diffuse vs. Localized Damage

**Figure 8:**
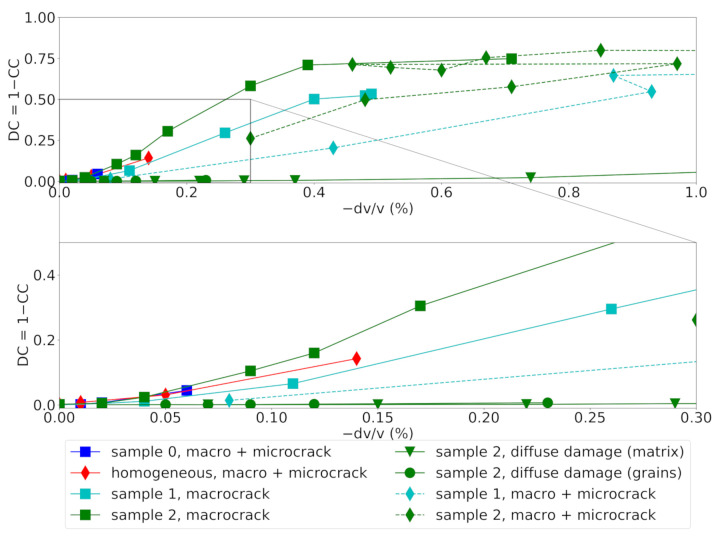
Decorrelation coefficient versus apparent velocity change for all different models and damage scenarios. Bottom: zoom-in view of the initial portion with small velocity changes.

The decorrelation coefficient ($DC=1-CC_{max}$) is plotted over the observed velocity change dv/v for all numerical experiments in Figure 8. The naming and coloring of scenarios is consistent with the previous two subsections.

In all cases, CWI recovers even very small velocity changes dv/v less than 0.1%. For the cases where constant diffuse damages is assumed a priori, the decorrelation coefficient remains low even for large changes in elastic parameters. If the velocities change evenly in the complete sample, the stretching of the waveforms can match the initial waveform. More localized changes in velocities caused by micro- and macrocracking lead to rapid deterioration of the correlation of the waveforms, as in this case, as localized damage introduces more complex changes in the waveform and the waveforms cannot be matched by simply stretching the signals anymore.

In case microcracking is suppressed in samples 1 and 2 (“macrocrack”), a steep increase of DC is observed already for small velocity changes dv/v. The evolution of the curves for samples 1 and 2 with a full transition from diffuse to localized damage (“micro+macrocrack”) shows a decorrelation trend between the one observed for localized macrocracks (“macrocrack”) and the scenarios of artificial diffuse damage in the mortar matrix and the grains in the complete specimen (“diffuse damage”). Diffuse microcracks occupy a larger part of the model than the macrocrack. Therefore, the trend of the decorrelation may be representative of the spatial volume involved in microcracking, in which the wave velocity is being reduced.

In this set up with a single source and a single receiver, the location of the macrocrack (closer to the source or closer to the receiver) produced nearly identical results due to the source-receiver reciprocity of the elasto-dynamic wave equation.

## 4. Discussion: Implications for Large-Scale Structural Monitoring

In the previous section, the capability of CWI to discriminate between different damage scenarios has been demonstrated. Further confirmation of the results of this study could be achieved by measuring temperature and moisture simultaneously with acoustic emission signals, i.e., with a system such as reported in [29]. Although the setup has been created as close to laboratory experiments as possible, some aspects should be discussed in view of a potential application of CWI for structural health monitoring of larger structures.

### 4.1. Numerical Concrete Models

The used approach to create mesostructural models has been demonstrated to produce very realistic results [17]. Similarly, the DEM method produces very realistic cracks [23] and may be used to investigate different loading scenarios on the same sample. A fact which is not possible in laboratory experiments. Future research may focus on introducing imaging procedures, such as microcomputer-tomography [23], to the workflow of the Concrete Mesostructure Generator to investigate specific concrete specimens in more detail.

### 4.2. Influence of Recording Length

**Figure 9:**
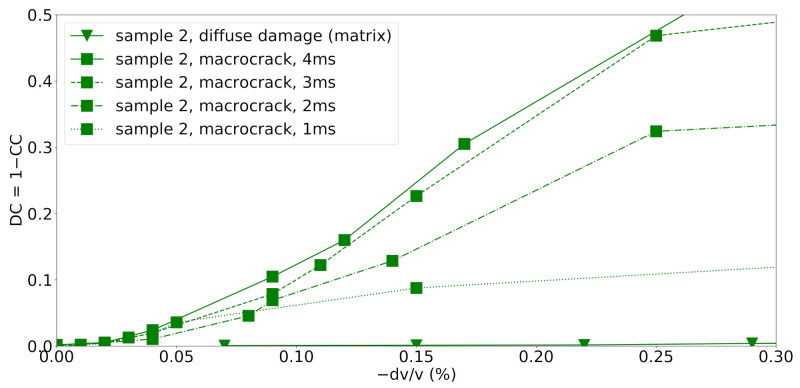
Decorrelation coefficient versus velocity change for different recording lengths for the macrocrack case in sample 2 with microcracking being suppressed and for a scenario with a constant diffuse damage distributed in the mortar matrix.

In the previous section, the recording length was fixed to 4ms. In practice, shorter recording times might be considered to save on total measurement times. Therefore, the CWI analaysis performed for the localized macrocrack in sample 2 is repeated with different recording lengths. The traces are cut after 1ms, 2ms and 3ms (Figure 9). For comparison, the scenario “diffuse damage (matrix)”, i.e., constant diffuse damage induced in the mortar matrix, is included in Figure 9 as well. With shorter recording times, it becomes more challenging to distinguish a localized damage from a diffuse damage (Figure 9). This is consistent with the theory of multiply scattered waves. For longer recording times, the waves scattered at more points and reflected multiple times at the boundaries of the model are included in the analysis. Thus, more information about the internal structure of the medium is included in the CWI analysis.

The figure also shows that a recording length of 4ms does not lead to an improvement with respect to a recording length of 3ms. The used source signal has a dominant frequency of $f_{c}=60\;\mathrm{kHz}$ and thus a dominant period of $T=\frac{1}{f_{c}}\approx 0.01667\;\mathrm{ms}$. Therefore, recording lengths of 1,2,3and4ms mean that 60,120,180and240 periods are recorded, respectively. Hence, a recording length that allows for at least 180 dominant periods to be recorded seems to be favorable to capture most information available from the coda.

In this study, the complete signal is used for the analysis. In practice it may be beneficial to exclude the first arrival and/or investigate different parts of the Coda signal separately to recover the velocity changes at different distances with respect to the transducers.

### 4.3. Influence of Boundary Conditions

**Figure 10:**
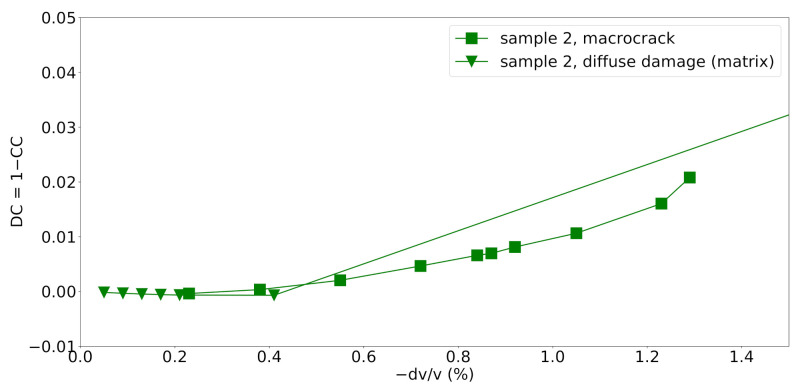
Decorrelation coefficient versus velocity change assuming absorbing boundary conditions obtained for sample 2 with microcracking being suppressed and for a scenario with a constant diffuse damage distributed in the mortar matrix.

In the previous simulations, vacuum boundaries are assumed around the sample to mimic finite-sized concrete specimen. If larger structures are monitored (e.g., bridges), the ultrasonic wave field travels only through parts of the structure. Depending on the transducer positions, the wave field is not reflected at the boundaries. Therefore, absorbing boundaries approximate this particular situation more adequately. Simulations for sample 2 with suppressed microcracking and the diffuse damage scenario in the mortar matrix are repeated with absorbing boundaries. It is hardly possible to distinguish the different damage scenarios (Figure 10). The decorrelation coefficients are far smaller than in the previous cases. The lack of reflections from the boundaries of the model significantly reduces the illumination of the damage. Furthermore, the obtained velocity changes are considerably smaller for the diffuse damage case and considerably larger for the macrocrack scenario as compared to the respective case assuming vacuum boundaries.

Further studies need to be performed to enhance and update the CWI workflow to make it suitable for large-scale structures. Preliminary ideas involve the use of a set of optimally positioned transducers [10] and using distributed fiber optic cables for significantly enlarged datasets [30] and incorporate strain measurements [31]. Furthermore, combining CWI with diffuse wavefield kernels [9] or other imaging techniques from passive seismology seem promising in overcoming this challenge. Another promising alternative has been proposed recently. In [32] the authors detect damages in materials using reflection signals instead of typical transmission setups such as investigated in this study.

## 5. Conclusions

The sensitivity of Coda Wave Interferometry to different damage scenarios has been investigated numerically. Realistic numerical concrete models were created with three different grain size ranges. The response of concrete specimen subjected to uniaxial tension has been simulated by means of the discrete element method to generate a set of virtual mesostructural concrete models with evolving microcracks, which are eventually coalescing into a macrocrack. Simulations of the ultrasonic wave propagation were performed on these models for all stages during tensile loading and compared to scenarios, where a constant diffuse damage has been assumed in the mortar matrix and the grains, respectively. The main conclusions of this study are as follows:

- The feasibility of CWI to detect small-scale velocity changes very early has been demonstrated.
- The decorrelation of wave forms indicates the spatial extent of the damage, enabling the detection of distributed microcracking in early stages of the loading, while a constant diffuse damage shows almost no change in the decorrelation coefficient.
- On the specimen level it was possible to differentiate between diffuse and localized damage using CWI.
- Comparing reflective and absorbing boundary conditions revealed with absorbing boundary conditions, it was no longer easily possible to distinguish localized and diffuse damage as the lack of reflections from the boundaries significantly reduces illumination of the damage.
- Applications of CWI on larger scale specimen should prioritize long recording lengths to monitor and discriminate multi-scale damages.
- The use of multiple optimally placed transducers seems to be crucial for structural health monitoring of infrastructures.

## Figures and Tables

**Table 1:** Statistical data of numerical concrete samples and minimum and maximum grain sizes ($a_{min},a_{max}$).

	Number of Grains	Volume Fraction (%)	$a_{\min}$ (mm)	$a_{\max}$ (mm)
Sample 0	15271	40.08%	4	16
Sample 1	2713	31.41%	10	20
Sample 2	915	31.11%	15	30

**Table 2:** Summary of the model parameters required in calibration of the DEM model.

Elastic parameters		Mortar	Aggregate	
$K_{n}$	normal modulus	8	16	GPa
$K_{\tau }$	tangential modulus	1	2	GPa
**Damage law in tension**	
$\varepsilon_{0}$	limit elastic strain	$1\times 10^{-4}$	$1\times 10^{-4}$	
$\frac{\varepsilon_{f}}{\varepsilon_{0}}$	relative ductility	5, 50	5, 50	
**Elasto-plasticity in shear**		
$c_{0}$	initial cohesion	1	2	MPa
tanϕ	frictional angle	0.57	0.57	

**Table 3:** Tensile strength, Elastic Modulus and Strain at peak load obtained from DEM simulations on 3 virtual concrete samples.

	Tensile Strength (MPa)	Elastic Modulus (GPa)	Strain to Peak Load (10-6)
**Sample 0**	1.51	17.89	82
**Sample 1**	2.24	16.59	157
**Sample 2**	1.91	16.63	144

**Table 4:** P-wave velocity $v_{p}$, S-wave velocity $v_{s}$, density ρ, elastic bulk moduli *K* and shear moduli μ used for the realistic numerical concrete model. The parameters for the matrix are taken from [24] and the values for the grains are approximated from [25].

	$v_{p}$ (m/s)	$v_{s}$ (m/s)	ρ ($\mathrm{kg}/m^{3}$)	*K* (GPa)	μ (GPa)
matrix	3950	2250	2050	18.147625	10.378125
grains	6230	3330	2950	70.881715	32.712255

**Table 5:** Relation between minimum and maximum grain sizes ($a_{min}$, $a_{max}$) in the three used samples and the dominant wavelength of the source signal, computed as $\lambda =\frac{v_{p}^{matrix}}{f_{c}}=\frac{3950\;m/s}{60\;\mathrm{kHz}}$.

	$a_{\min}$ (mm)	$a_{\max}$ (mm)	$\frac{a_{\min}}{\lambda }$ (%)	$\frac{a_{\max}}{\lambda }$ (%)
sample 0	4	16	6.1	24.3
sample 1	10	20	15.2	30.4
sample 2	15	30	22.8	45.6

## Data Availability

The data are available upon request from the authors.
